# Leveraging Larger AES Keys in LoRaWAN: A Practical Evaluation of Energy and Time Costs

**DOI:** 10.3390/s23229172

**Published:** 2023-11-14

**Authors:** Phithak Thaenkaew, Bruno Quoitin, Ahmed Meddahi

**Affiliations:** 1 Department of Computer Science, University of Mons, 7000 Mons, Belgium; phithak.thaenkaew@umons.ac.be; 2 Centre for Digital Systems, IMT Nord Europe, Institut Mines-Télécom, 59000 Lille, France; ahmed.meddahi@imt-nord-europe.fr

**Keywords:** LoRaWAN, IoT security, Advanced Encryption Standard (AES)

## Abstract

Internet of Things (IoT) devices increasingly contribute to critical infrastructures, necessitating robust security measures. LoRaWAN, a low-power IoT network, employs the Advanced Encryption Standard (AES) with a 128-bit key for encryption and integrity, balancing efficiency and security. As computational capabilities of devices advance and recommendations for stronger encryption, such as AES-256, emerge, the implications of using longer AES keys (192 and 256 bits) on LoRaWAN devices’ energy consumption and processing time become crucial. Despite the significance of the topic, there is a lack of research on the implications of using larger AES keys in real-world LoRaWAN settings. To address this gap, we perform extensive tests in a real-world LoRaWAN environment, modifying the source code of both a LoRaWAN end device and open-source server stack to incorporate larger AES keys. Our results show that, while larger AES keys increase both energy consumption and processing time, these increments are minimal compared to the time on air. Specifically, for the maximum payload size we used, when comparing AES-256 to AES-128, the additional computational time and energy are, respectively, 750 ms and 236 μJ. However, in terms of time on air costs, these increases represent just 0.2% and 0.13%, respectively. Our observations confirm our intuition that the increased costs correlate to the number of rounds of AES computation. Moreover, we formulate a mathematical model to predict the impact of longer AES keys on processing time, which further supports our empirical findings. These results suggest that implementing longer AES keys in LoRaWAN is a practical solution enhancing its security strength while not significantly impacting energy consumption or processing time.

## 1. Introduction

The Internet of Things (IoT) has shown rapid growth and versatility in recent years, facilitating numerous applications across a wide range of fields, including smart homes, health care, industrial automation, and more [1]. A crucial element of IoT is the communication between devices, especially in scenarios requiring long-range, low-power wireless communication. In this regard, LoRaWAN (Low Range Wide Area Network) is a widely adopted protocol, providing bi-directional communication that supports a large number of devices over a wide area. It is particularly suited for battery-powered devices due to its low power consumption [2,3].

Despite the robustness and growing popularity of LoRaWAN, various potential vulnerabilities in the protocol’s security implementation have been identified. A 2018 study discovered and analyzed several security vulnerabilities in LoRaWAN, including replay attacks, plaintext recovery, malicious message modification, falsification of delivery reports, and battery exhaustion attacks [4]. Furthermore, a 2017 investigation highlighted vulnerabilities in the LoRaWAN join procedure, potentially enabling denial of service (DoS) attacks [5].

LoRaWAN’s security significantly relies on the Advanced Encryption Standard (AES) to protect the confidentiality and integrity of the transmitted data [2,3]. This symmetric key algorithm comes in three versions: AES-128, AES-192, and AES-256, where the numerical suffix indicates the bit length of the cryptographic key. The primary difference between these versions lies in the number of rounds of the encryption process: 10 rounds for AES-128, 12 for AES-192, and 14 for AES-256 [6]. As a result, variants of AES with more encryption rounds and larger key sizes, such as AES-256, are theoretically more secure but would require more computational resources compared to AES-128 [7,8].

Parallel to other advancements in the technology landscape, there has been an exponential increase in supercomputing capabilities, which further amplifies these threats. For instance, the leading supercomputer’s speed, which was 59.70 gigaflops in 1993, soared to 35,860.00 gigaflops in 2003, 33,862.70 teraflops in 2013, and 1194.00 petaflops in 2023 [9]. These figures suggest that a brute-force attack on AES-128, theoretically requiring around $3.4\times 10^{38}$ attempts to break, may not remain impractical forever given the increasing computing power.

While traditional supercomputing capabilities continue to grow, the emerging field of quantum computing poses an even more pressing threat to the security of the AES, particularly AES-128, which is currently used in LoRaWAN. Quantum computers are anticipated to break modern public key cryptography due to Shor’s algorithm [10], necessitating a transition to quantum-resistant algorithms [11]. In 1996, Grover’s algorithm was proposed, which could potentially search a database with square root time complexity [12]. Then, in 2016, the application of Grover’s algorithm to AES was examined, providing resource estimates for potential quantum attacks [13].

In light of the rapidly advancing quantum technology capabilities, the urgency of transitioning to quantum-resistant algorithms is increasingly apparent. The Quantum Roadmap released by IBM underscores this urgency, highlighting the increase in the number of qubits in their quantum processors, from 27 qubits in 2019 to an anticipated 1121 qubits by 2023, and further scaling to 10,000–100,000 qubits by 2026 or later [14]. The roadmap indicates an accelerating pace of quantum computing development, including the advent of advanced multi-chip quantum processors and quantum communication technologies.

The concept of a store-now-decrypt-later (SNDL) attack presents another major threat. In this scenario, adversaries capture valuable encrypted information now, store it, and decrypt it later once large fault-tolerant (LFT) quantum computers are available [11]. This underscores the need for proactive security measures in anticipation of these developments.

The National Security Agency (NSA) has underscored the importance of planning and transitioning towards quantum-resistant algorithms in its guidance on future quantum-resistant algorithm requirements [15]. Notably, they recommend the use of 256-bit keys for all classification levels, offering a higher level of security than AES-128 [16]. This situation highlights the need for exploring longer key lengths, such as AES-256, to assure the sustained protection of IoT communications in the future. However, the application of AES-256 in the LoRaWAN protocol presents challenges related to power consumption and processing time.

Given the continuous growth in computing power, the more resource-intensive AES-192 and AES-256 could provide stronger security. However, their use in resource-constrained IoT devices, such as those utilizing the LoRaWAN protocol, presents challenges due to increased power consumption and processing time. As noted by Li et al. (2019), these devices are often not designed with robust security features in mind and hence pose significant challenges regarding privacy and security. This underscores the importance of maintaining a careful balance between security and resource efficiency in the context of IoT communications [17].

This paper aims to study the feasibility of employing the longer AES keys, specifically AES-192 and AES-256, in the LoRaWAN protocol, considering their energy consumption, processing time, and security benefits compared to the currently used AES-128. Notably, prior to our work, there has been no research conducted in a real practical LoRaWAN environment addressing this topic. In addition to studying the impact on performance, our research highlights the fact that using larger keys with AES is a realistic solution for enhancing LoRaWAN security that can be deployed in the very short term, requiring only limited software updates to end devices and servers.

The remainder of this paper is organized as follows: Section 2 provides a review of the related work in this field. In Section 3, we delve into the background information that is necessary for understanding the context of our study. Section 4 presents a mathematical model of AES encryption and integrity as defined in LoRaWAN, which we later confront in our experimental results. The test-bed and methodology used in our experiments are explained in Section 5. Section 6 presents the results of our study. We discuss these results and their implications in Section 7.

## 2. Related Work

This section presents a synopsis of previous research that examines LoRaWAN security, specifically its correlation with power consumption.

Despite the lack of explicit recommendations for enhancing the security of the network core within the LoRaWAN standard, innovative secure LoRaWAN structures have been suggested [18,19]. Observing the significant vulnerability of the gateway due to its placement outside the network core, Oniga and associates [18] proposed linking the gateways to the core through VPNs. To ensure internal security within the network, such as between network and application servers, they used TLS and mutual authentication grounded on a Public Key Infrastructure (PKI) and Certificate Authority (CA). An alternate secure LoRaWAN backend [19] focuses on the communication layer between stack servers. Their proposition, known as Server Session Key Generation (S2KG), employs Elliptic Curve Cryptography (ECC) to derive network session keys.

Several frameworks have been proposed for evaluating LoRaWAN security. For instance, ChirpOTLE [20] was used to assess a unique adaptive data rate (ADR) spoofing attack and to pinpoint the susceptibility related to the lack of beacon authentication in Class B operation. These discoveries led the authors to suggest amendments to the LoRaWAN specification to mitigate such attacks. Another framework [21] merges standalone LoRaWAN transceivers with software-defined radio (SDR) and GNU Radio, enabling them to simulate a man-in-the-middle attack.

Several assessments have delved into symmetric-key cryptography on devices with limited resources. For instance, a study by Rif‘a-Pous and Herrera-Joancomartí [22] evaluated the performance of DES, 3DES, and AES on PDAs. Another study [23] contrasted the processing time of multiple lightweight hardware substitution–permutation network (SPN) block ciphers, intending to implement them on devices with fewer resources. Research on AES latency and energy consumption on Contiki-based IoT devices was also carried out [24], utilizing the Texas Instruments ARM-based CC2650 to test three AES software  implementations. They found that Contiki’s inbuilt AES performed better in terms of both energy consumption and processing time. A comparative evaluation using a similar platform, the Texas Instruments CC1310, showed that hardware implementations of AES effectively reduce processing time and energy consumption [25]. However, the software still plays a vital role in applications across multiple blocks. A low-power AES data encryption structure, LPADA [26], has been proposed for LoRaWAN. This approach involves using a low-power lookup table for AES substitution and better power distribution management of unused AES logic during the various rounds of the AES encryption process.

Another theme in LoRaWAN security research is the application of asymmetric key cryptography. Mårlind and Butun [27] put forth a new activation process for LoRaWAN end devices leveraging public key cryptography (PKC). They termed this process as public key over-the-air activation (PK-OTAA), which enables devices to dynamically acquire root keys that can be altered when necessary. The use of PKC calls for larger keys, hence increasing the demand for processing power and consequently raising battery consumption. However, the authors identified that this trade-off provides a key distribution scheme that offers improved flexibility and security. In a similar vein, Milani and Chatzigiannakis [28] explored the shortcomings of the current over-the-air activation (OTAA) technique in LoRaWAN architecture. They underscored that the inability to update the network key (NwkKey) and application key (AppKey) over a deployment’s lifetime, often spanning over ten years, creates a security loophole. To address these vulnerabilities, they introduced a network activation scheme that securely negotiates new keys periodically, relying on ECC. This technique successfully mitigates certain vulnerabilities while preserving backward compatibility. Both these studies [27,28] mark significant advancements in the utilization of asymmetric key cryptography in LoRaWAN security, although the energy impacts of these methods need further examination.

A theoretical model predicting the energy performance of LoRaWAN was also put forth [29]. The study projected that an end device running on a 2400 mAh battery with a 5-min message transmission interval could achieve a battery life of one year, potentially extending to six years with longer intervals. However, the cost of security primitives was not a focus of this study.

Additionally, Winderickx et al. [30] conducted a detailed analysis of the energy consumption associated with implementing various security algorithms on IoT devices. Their investigation spanned multiple platforms and wireless communication protocols, including BLE, Wi-Fi, and LoRaWAN. They meticulously studied both computation and communication energy costs, leading them to formulate an equation for calculating the minimal session duration that would balance power consumption and side-channel resistance. The insights derived from their work offer valuable guidance in choosing suitable security algorithms and wireless communication protocols while also shedding light on factors influencing the overall energy consumption in IoT applications.

In the initial phase of this research, a preliminary study [31] was conducted to examine the implications of using different AES key lengths on a LoRaWAN end device. At the time of this initial exploration, there was no existing research probing into the consequences of using longer AES key lengths within the LoRaWAN architecture. Our experiment involved executing primitive encryption functions with a variety of key and payload sizes, allowing us to observe the associated differences in energy consumption and processing time. The results indicated that the use of longer keys had a relatively minor theoretical impact on these factors when compared to the costs from other tasks, thereby suggesting their potential suitability for improving LoRaWAN security. However, the scope of the study was limited to the primitive encryption function on the end device and did not extend to investigating the implementation of longer keys across the entire LoRaWAN infrastructure. Moreover, it did not offer an in-depth model of the energy and computational time consumption associated with the deployment of longer AES keys. Our current research seeks to address these gaps by investigating the practical application of these concepts in a real-world LoRaWAN environment.

## 3. Background

This section provides comprehensive background on various key aspects relevant to our study. Firstly, an overview of LoRaWAN is presented to acquaint the reader with the basics of this low-power, wide-area networking protocol. We then delve into LoRaWAN security, focusing on the mechanisms that safeguard data integrity and confidentiality. Over-the-air activation (OTAA) is discussed as the predominant method for secure device initialization in LoRaWAN networks. The section also explains the cryptographic algorithms used in LoRaWAN, such as AES, AES-CCM*, and AES-CMAC. Finally, we explore time on air (ToA), a crucial parameter that impacts the efficiency and scalability of the network. Each of these subtopics is vital for a comprehensive understanding of the performance and security implications in LoRaWAN environments.

### 3.1. Overview of LoRaWAN

Long Range Wide Area Network (LoRaWAN) is a popular low-power wide-area network (LPWAN) protocol designed to wirelessly connect battery-operated devices in regional, national, or global networks. It targets key requirements of the Internet of Things, such as secure bi-directional communication, mobility, and localization services. This standard is developed by the LoRa Alliance, a non-profit association committed to enabling large-scale deployment of Low Power Wide Area Networks (LPWANs) for IoT through the development and promotion of the LoRaWAN open standard.

**Figure 1:**
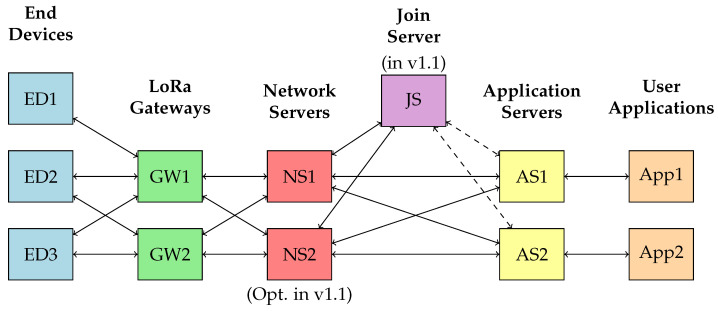
LoRaWAN network architecture.

The LoRaWAN architecture, as shown in Figure 1, comprises several components, including end devices, LoRa gateways, network servers, application servers, and applications. Each component plays a unique role in the network:

- *End Devices (EDs)*: These are IoT devices like sensors or actuators that collect data and transmit them to the LoRa gateways. The devices are usually located remotely and operate on battery power.
- *LoRa Gateways (GWs)*: The gateways act as a bridge between EDs and the network servers. They receive data transmitted by EDs and forward them to the network servers.
- *Network Servers (NSs)*: The network servers manage the network, including handling the routing of messages to the appropriate application servers and managing the adaptive data rate (ADR) for EDs. In the LoRaWAN v1.1 architecture, there is an option for having more than one network server for enhanced performance and reliability.
- *Join Server (JSs)*: Introduced in LoRaWAN v1.1, the join server is responsible for securely handling the initial activation process for EDs, providing a higher level of security.
- *Application Servers (ASs)*: Application servers handle the application-specific logic, processing the data received from EDs, and optionally sending data back to the devices.
- *User Applications* (Apps): These are the end-user applications that utilize the data collected and processed by the network.

Each of these components has its unique energy consumption and security considerations, which we will discuss in the following sections. It is important to note that the focus of this paper is on LoRaWAN v1.0. In this version, the authentication process is carried out through NS, rather than JS introduced in later versions like LoRaWAN v1.1.

### 3.2. LoRaWAN Security

LoRaWAN’s network security is an integral part of its architecture, providing protection for the information being transmitted across the network. Two encryption keys are implemented in the network: one for the network (Network Session Key, NwkSKey) and one for the application (Application Session Key, AppSKey). Both of these keys employ AES with a key size of 128 bits.

The security provided by LoRaWAN is essential for ensuring the confidentiality, authenticity, and integrity of the data being communicated. Confidentiality is achieved by encrypting the data payload with the AppSKey, preventing unauthorized access to the content of the data. Authenticity and integrity are ensured by using a message integrity code (MIC) computed with the NwkSKey, validating the source of the message and confirming that it has not been tampered with during transmission.

The LoRaWAN version 1.1 specification introduced several enhancements to address identified security issues and potential vulnerabilities in version 1.0. Among the major improvements in version 1.1 are the addition of a JS, as shown in Figure 1, and the introduction of end-device-specific security keys for NSs and ASs. This latter change allows each server to have its own set of keys, significantly improving the security of the network against potential attacks. However, both versions of the standard continue to employ AES-128 as the basis for their encryption mechanisms, a choice driven primarily by a balance of security needs and resource constraints in IoT devices.

It is important to note that, while version 1.1 provides more robust security, it also comes with added implementation complexity. For this study, we elected to base our experiments on LoRaWAN version 1.0 due to its more straightforward implementation. We contend that the results of our investigation are still applicable to version 1.1 given the commonality of the encryption method. Despite the enhancements in security management, the fundamental operation of the AES encryption in the LoRaWAN protocol remains consistent between versions 1.0 and 1.1.

The next section delves into the specifics of AES and its utilization in the LoRaWAN architecture.

### 3.3. Over-the-Air Activation (OTAA)

**Figure 2:**
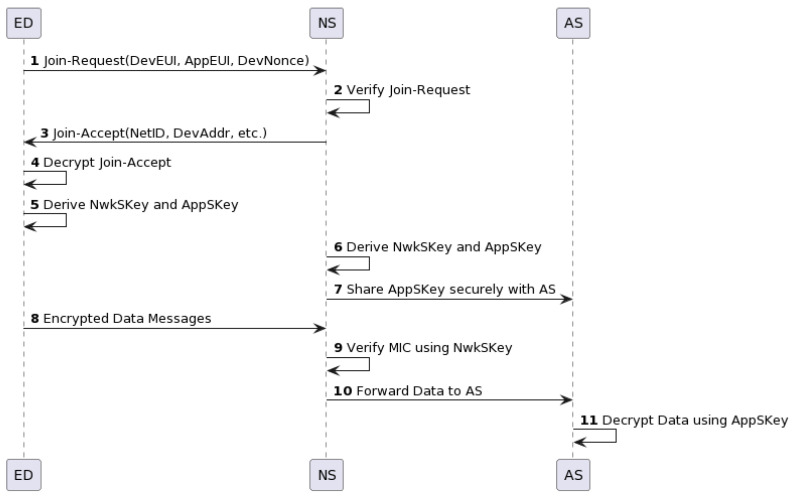
LoRaWAN v1.0 over-the-air activation.

Figure 2 illustrates the OTAA process in LoRaWAN version 1.0. As shown in the diagram, ED initiates the process by sending a Join-Request to NS. This request includes the DevEUI (a unique identifier for the device), the AppEUI (application identifier), and the DevNonce (a device nonce to ensure request freshness). NS then verifies the request using the AppKey,  a pre-shared key between ED and NS.

Upon successful verification, NS sends a Join-Accept message to ED, which includes the network identifier (NetID), device address (DevAddr), and other parameters necessary for network communication. ED decrypts this message using the AppKey and then uses the received parameters to derive two session keys: the network session key (NwkSKey) and the application session key (AppSKey).

At the same time, NS derives the identical session keys and securely shares the AppSKey with AS. With these session keys in place, ED can start sending encrypted data messages to NS. NS verifies the integrity of these messages using the NwkSKey and then forwards the encrypted data to AS. AS uses the AppSKey to decrypt these data and extracts the information sent by ED.

In essence, the OTAA process in LoRaWAN v1.0 involves a single exchange of Join-Request and Join-Accept messages, leading to the derivation and deployment of session keys. However, it is important to note that LoRaWAN v1.1 introduces enhancements to this process, including an extra layer of security with JS and the introduction of additional session keys for increased security granularity.

In the following section, we delve into the details of AES, its role in the LoRaWAN security scheme, and how different AES key lengths could affect the energy efficiency and security of LoRaWAN networks.

### 3.4. Advanced Encryption Standard (AES)

The Advanced Encryption Standard (AES) is a symmetric key encryption standard established by the US National Institute of Standards and Technology (NIST) in 2001 [6]. AES is based on a block cipher known as Rijndael, and it operates on data blocks of 128 bits using keys of lengths 128, 192, or 256 bits.

AES works on the principle of substitution and permutation, performing several rounds of these operations to convert plaintext into ciphertext. For a 128-bit key, AES performs 10 rounds, while, for 192- and 256-bit keys, it performs 12 and 14 rounds, respectively.

In the context of LoRaWAN, AES is used for encryption and integrity checking. For the encryption part, LoRaWAN uses the AES-Counter with CBC-MAC (AES-CCM*) mode. This mode combines the Counter (CTR) mode for encryption and the Cipher Block Chaining Message Authentication Code (CBC-MAC) mode for integrity checking.

In the next sections, we explore more about the use of AES in LoRaWAN, specifically how AES-CCM* and AES-CMAC, two AES-based methods, are used for data encryption and integrity checking, respectively.

### 3.5. AES-CCM*

AES-CCM, defined by the NIST standard [32], is a block cipher mode that combines the Counter mode (CTR) for ensuring confidentiality and the CBC-MAC mode for authentication. A variant known as AES-CCM* is adapted from the IEEE 802.15.4 standard [33] and offers the flexibility of being used solely for encryption. In both AES-CCM and its variant, the same key is used for both encryption and authentication processes.

**Figure 3:**
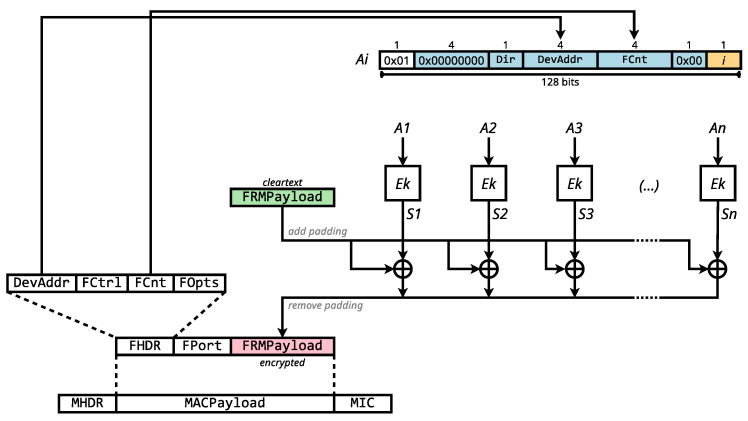
LoRaWAN AES-CCM* encryption transformation.

As illustrated in Figure 3, AES-CCM* operates by constructing a sequence of blocks, $A_{i}$, centered around a unique nonce and a counter *i*. This nonce is generated using message header fields like DevAddr and FCnt. Each $A_{i}$ block is encrypted with the AES cipher $E_{k}$ to yield $S_{i}$, which is subsequently XORed with its corresponding plaintext block to produce the ciphertext. If the plaintext’s length is not a multiple of 128 bits, zero-padding is applied.

### 3.6. AES-CMAC

AES-CMAC is employed in LoRaWAN primarily for data origin authentication and integrity during the over-the-air activation (OTAA) process. In OTAA, ED initiates a network connection by transmitting a Join-Request, comprising fields such as the device’s unique identifier (DevEUI), application identifier (AppEUI), and a device nonce (DevNonce).

Crucially, the Join-Request incorporates an MIC computed via AES-CMAC, using the device’s pre-shared AppKey. This MIC allows NS to confirm the message’s integrity and origin. The server’s reply—a Join-Accept message—similarly includes an MIC, calculated using its own AppKey, enabling ED to perform a similar validation.

**Figure 4:**
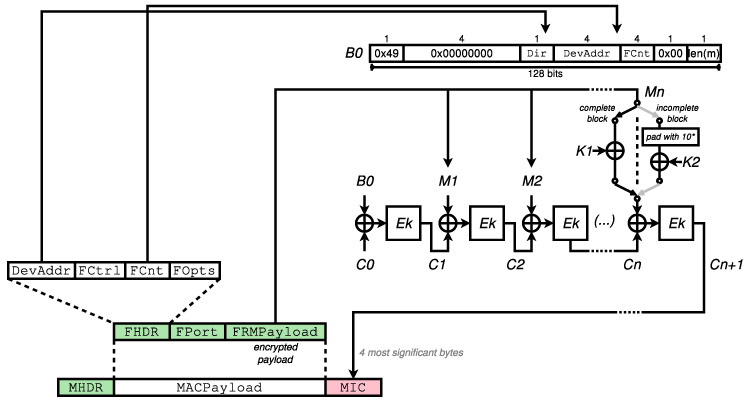
LoRaWAN AES-CMAC applied to a data frame.

As illustrated in Figure 4, the message is composed of the  MHDR, FHDR, FPort, and FRMPayload fields. $K_{1}$ and $K_{2}$ are subkeys generated from the session key to be used. CBC is applied using an initial value of $C_{0}$, first to the special block $B_{0}$, then on each block from the message. The last block $M_{n}$ of the message is either XORed with $K_{1}$ or padded, then XORed with $K_{2}$ prior to being processed, depending on $M_{n}$ being a complete block, that is having a size of exactly 128 bits, or not. Note that 10* in the figure denotes padding with a leading bit equal to 1 followed by the required number of 0, as prescribed by the AES-CMAC specification.

With the key security aspects of LoRaWAN now elucidated, we move on to another vital operational feature: time on air.

### 3.7. Time on Air (ToA)

A critical consideration for any wireless communication system, especially those focusing on low power and long range, such as LoRaWAN, is the “time on air” (ToA). ToA is the duration a signal spends occupying the wireless medium for a single transmission. The duration of this transmission can have direct implications on the power consumption of a device: a longer transmission time can increase power consumption. Additionally, a longer ToA can reduce network capacity as a channel is occupied for a longer period, reducing its availability for other transmissions. Understanding and calculating ToA in LoRaWAN involves a number of parameters and steps, as described in detail by the Semtech Corporation’s “LoRa Modem Designer’s Guide” [34].

The ToA in LoRaWAN can be computed using the following equations:

1. Symbol Duration ($T_{symbol}$): The duration of a symbol is calculated using the spreading factor (SF) and bandwidth (BW) as provided by
  (1) $$T_{symbol}=\frac{2^{SF}}{BW}$$
2. Preamble Duration ($T_{preamble}$): The duration of the preamble is determined by the preamble length parameter (usually 8) and the symbol duration:
  (2) $$T_{preamble}=\left(PreambleLength+4.25\right)\times T_{symbol}$$
3. Payload Symbol Number (PSymbNb): The number of payload symbols can be calculated considering various factors like payload length (PLength), spreading factor (SF), implicit header (IH), data rate optimization (DE), and coding rate (CR):
  (3) $$PSymbNb=8+\max\left(\left\lceil \frac{8\times PLength-4\;\cdot \;SF+28+16-20\;\cdot \;IH}{4\times (SF-2\;\cdot \;DE)}\right\rceil \times \left(CR+4\right),0\right)$$
4. Payload Duration ($T_{payload}$): The duration of the payload is determined by the payload symbol number and the symbol duration:
  (4) $$T_{payload}=PSymbNb\times T_{symbol}$$
5. Time on Air (ToA): Finally, the total time on air can be calculated by summing the preamble and payload durations:
  (5) $$ToA=T_{preamble}+T_{payload}$$

ToA calculation is particularly influenced by the modulation parameters of LoRaWAN specified for various regions. Taking the EU region as an example, typical modulation parameters are a spreading factor (SF) of 7, a bandwidth (BW) of 125 kHz, a preamble length of 8, and a maximum payload length of 235 bytes (comprising 222 bytes for the payload and 13 bytes for headers). Furthermore, we take into account a code rate (CR) of 4/5, which is represented by a value of 1 in our system settings. Other pertinent parameters, such as implicit header (IH) and data rate optimization (DE), are also factored in, both being represented by a value of 0 in our calculations.

Given these specific LoRaWAN modulation parameters for the EU region, we can illustrate the ToA calculation for a packet. Thus, with the mentioned parameters in play, especially an SF of 7 and a payload length of 235 bytes, the time on air (ToA) for the transmission amounts to approximately 368.9 ms.

It is important to note that parameters like the payload length can significantly influence the ToA. For instance, an increased payload length would increase the ToA, which in turn could affect the energy consumption and efficiency of LoRaWAN devices. This ties back into the main focus of our research: understanding the impact of varying AES key sizes on energy consumption and processing time within LoRaWAN. In the following sections, we develop a mathematical model and conduct experiments to explore this aspect further.

## 4. Mathematical Model

The aim of this paper is to study the impact of using longer AES keys (192 and 256 bits) on the AES-CCM* and AES-CMAC implementations as used in LoRaWAN. In this section, we construct a mathematical model to quantify the computational overhead introduced by using larger AES key sizes compared to the standard 128-bit key. The differential time cost, represented as ΔT, provides insights into the added computational costs when selecting for stronger encryption techniques. We apply this model to two essential operations: encrypt_payload() and compute_mic(). This model is used as a basis to validate the empirical results obtained in our test-bed experiments.

### 4.1. Function encrypt_payload()

The encrypt_payload() function is responsible for encrypting the payload data, as explained in Section 3.5. The complete time duration, *T*, contains two primary elements: the time taken during the AES process iterating over the number of data blocks and static overheads, which include initialization and cleanup tasks.

To determine the number of blocks, $N_{b}$, needed for a payload of *N* bytes, Equation (6) is used, where 16 at the denominator is the size of an individual AES block, in bytes. Note that we round up the result to take account of the fact that AES encryption is applied over a full 128-bits block, with zero-padding being applied if necessary.

(6) $$N_{b}=\left\lceil \frac{N}{16}\right\rceil$$

The overall time duration of the encryption function is provided by Equation (7), where $T_{\mathrm{static}}$ aggregates the time consumed in initialization, cleanup, and other consistent-time overheads. The value of $T_{\mathrm{aes}\_1\_\mathrm{block}}$, the time required for AES processing of a single block of data, and $T_{\mathrm{static}}$ will be derived from experimental measurements.

(7) $$T=\left(N_{b}\times T_{\mathrm{aes}\_1\_\mathrm{block}}\right)+T_{\mathrm{static}}$$

It is essential to note that the time taken for AES processing of a single block, represented as $T_{\mathrm{aes}\_1\_\mathrm{block}}$, is influenced by the number of operation rounds in the AES algorithm. Specifically, AES with a 256-bit key requires 14 rounds, AES with a 192-bit key necessitates 12 rounds, and AES with a 128-bit key demands 10 rounds. Given the increasing number of rounds with longer key lengths, we can infer

(8) $$T_{\mathrm{aes}\_1\_\mathrm{block}256}>T_{\mathrm{aes}\_1\_\mathrm{block}192}>T_{\mathrm{aes}\_1\_\mathrm{block}128}$$

This inequality reflects the natural progression of computational time due to the increasing complexity of the encryption process with longer keys.

The additional time cost, ΔT, when adopting for larger AES key sizes (192 and 256 bits) versus the standard 128-bit key serves as a metric for measuring computational overhead.

(9) $$\Delta T=T_{K}-T_{128}$$

In Equation (9), *K* belongs to the set of AES key sizes {128, 192, 256}, and $T_{K}$ represents the time duration corresponding to the chosen AES key size.

### 4.2. Function compute_mic()

The compute_mic() function calculates the message integrity code (MIC) for a particular payload and mirrors the encryption process, as described in Section 3.6. However, an additional 9-byte header must be accounted for in the block number computation.

(10) $$N_{b}=\left\lceil \frac{N+9}{16}\right\rceil$$

The cumulative time taken by compute_mic() is effectively represented by *T*, as outlined in Equation (7), coupled with the extra headers for the MIC computation. The increased time when employing various key sizes, ΔT, can be determined using Equation (9).

Having constructed our mathematical models, we now transition to the Materials and Methods section, where we detail the experimental design used to validate our model.

## 5. Materials and Methods

To carry out our evaluation, we modified real LoRaWAN implementations to add support for longer AES keys during encryption/decryption and during message integrity code calculation. We have also instrumented the code for the purpose of establishing benchmarks. This involved modifying the AES-CCM* and AES-CMAC implementations. Two existing open-source LoRaWAN stacks were modified, one for the end device side and another one for the server side. In our implementation, we opted to have the size of the AES key to be used decided statically at compile time. Changing the key size requires a firmware update.

### 5.1. LoRaWAN Test Bed

**Figure 5:**
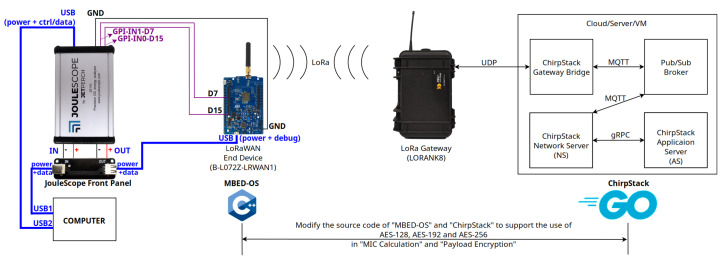
LoRaWAN environment and experiment setup.

Our measurement campaign was carried out over a real-world LoRaWAN environment composed of three core elements: EDs, GWs, and LoRaWAN servers. Figure 5 provides a visual representation of our LoRaWAN setup. In the figure, the left-hand side highlights the measurement component of our experiment, while the center and right-hand sides show the LoRaWAN network.

The ED deployed for the experiments was the B-L072Z-LRWAN1 Discovery Kit, an ARM-based development board equipped with a LoRa transceiver. It operates under the mbed-os platform, version 6.16, developed using the C++ programming language. mbed-os provides the necessary operating environment to develop the ED application firmware. As LoRaWAN gateway, we used the LORANK8 model. It relies upon the Semtech UDP packet forwarder protocol to communicate with the LoRaWAN server over an IP network. The server was deployed using the open-source ChirpStack project. It consists of a gateway bridge, an MQTT broker, a network server (NS), and an application server (AS). Chirpstack is developed using the Go programming language.

The following settings were used for the LoRa communications. We utilized the LoRaWAN 1.0.2 protocol. The spreading factor was set to SF7, the bandwidth was set to 125 kHz, and the adaptive data rate (ADR) scheme was enabled. The specific frequency plan adopted in our experiment was EU863-870, following the standard for LoRaWAN operations in the European Union. For each run of our experiment, the over-the-air activation (OTAA) was used for the registration process of the ED. After OTAA completion, the ED sent consecutive data messages to the LoRaWAN server with increasing payload sizes. Data messages were sent using the unconfirmed message type.

### 5.2. Measurement Methodology

Key to our experiment was the modification of the source codes of mbed-os [35] and specific components of ChirpStack, namely the network server [36] and application server [37], to support AES key sizes of 128, 192, and 256 bits. Specifically, the parts of the source code that were modified pertained to data encryption and MIC calculation. Both the ED and the LoRaWAN server were configured to use fixed keys for AES-192 and AES-256 to simplify implementation. Notably, these alterations required no hardware changes, thus preserving the physical configuration of our EDs.

In our tests, we aimed to measure the impact of different AES key sizes (128, 192, and 256 bits) on the energy consumption and computational time duration during the operations of encrypt_payload() and compute_mic(). For each experiment, the ED transmitted data to the LoRaWAN server with a payload size ranging from 1 to 222 bytes—the maximum payload size for the EU863-870 frequency band with SF7 [38]. Each payload size was tested ten times, allowing us to calculate average values and determine the range of results.

To measure the energy consumption of the ED and the time duration during the operations of encrypt_payload() and compute_mic(), we used JouleScope JS110, a precision DC energy analyzer. Two USB cables connected the computer to JouleScope, one at the back for controlling the measurement, and another at the front panel for the energy consumption measurement of the ED. Digital signals were sent from specific pins of the ED to trigger JouleScope to start and stop the energy consumption measurements.

Additionally, we used the mbed-os Timer class to measure the computational time usage of the ED during the encrypt_payload() and compute_mic() functions. These data were logged and later analyzed alongside the energy consumption data to form a comprehensive understanding of the impact of different AES key sizes on LoRaWAN device performance.

We conducted an analysis on the collected data, focusing on the increases in energy and computational time consumption due to the use of longer AES key sizes compared to the standard key size. In particular, we considered how these increases affected the encryption tasks of the ED in terms of data transmission for a single message.

By employing JouleScope, we were able to accurately monitor and manage the energy consumption during the operation of the encrypt_payload() and compute_mic() functions. The device’s ability to trigger measurements in sync with the ED’s operation significantly improved the precision of our energy consumption measurements.

In a complementary approach, we also utilized JouleScope to capture the measurements of the ED from power-on until the transmission of the first data uplink message. This step enabled us to study the behavior of various tasks, including the transmission of Join-Request and Join-Accept messages in OTAA, as well as the time on air (ToA). These data were instrumental in providing a complete picture of the system’s performance and behavior.

## 6. Results

Before delving into detailed results on the effects of AES key sizes and encryption functions, it is crucial to first grasp the fundamental behaviors of LoRaWAN communication. Hence, we undertook an in-depth examination of energy consumption during typical LoRaWAN operations.

**Figure 6:**
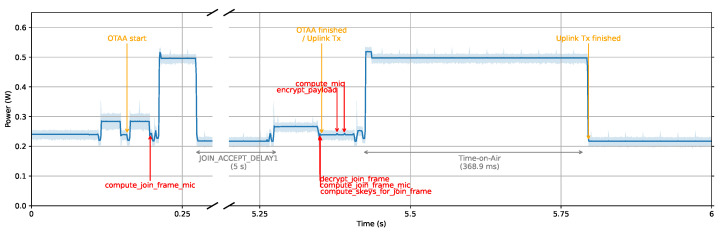
JouleScope trace of 222-byte data transmission with AES-128 including OTAA.

Figure 6 tracks the energy consumption from the moment ED powers on to the first uplink message’s transmission. This message carried a 222-byte payload and used AES-128 for LoRaWAN communication encryption. The x-axis represents time, while the y-axis indicates ED’s power usage.

This graph illustrates several key phases in the LoRaWAN communication process. The initial segment of the graph highlights the Join-Request process, marking the beginning of the OTAA authentication. This is followed by the Join-Accept portion, signaling the conclusion of the OTAA phase. Subsequently, the graph showcases the uplink of the first data message, with its peak power values indicating the time on air. A remarkable observation here is the significant power consumption during the data uplink, especially for the 222 bytes payload. This peak power consumption aligns with the time on air, spanning a duration of approximately 368.9 milliseconds. This empirically observed value aligns with our theoretical calculations from Section 3.7, using Equations (1)–(5) and our experiment’s specific parameters. During the time on air, we recorded an energy consumption of 183.703 millijoules as per the JouleScope measurements.

Having provided this foundational understanding of LoRaWAN’s behavior, we shall now delve into our core experimental findings. These experiments encompassed varying the AES key size (128, 192, and 256 bits), deploying encryption functions encrypt_payload() for AES-CCM* and compute_mic() for AES-CMAC, and manipulating the payload size within a range of 1 to 222 bytes. Four distinct graphs elucidate these findings, with each graph zeroing in on either the energy consumption or the processing time under specific conditions. Each of these graphs is labeled to reflect the measurement type and the function under test. The ensuing sections provide detailed expositions of each graph.

### 6.1. Measured Energy Consumption

**Figure 7:**
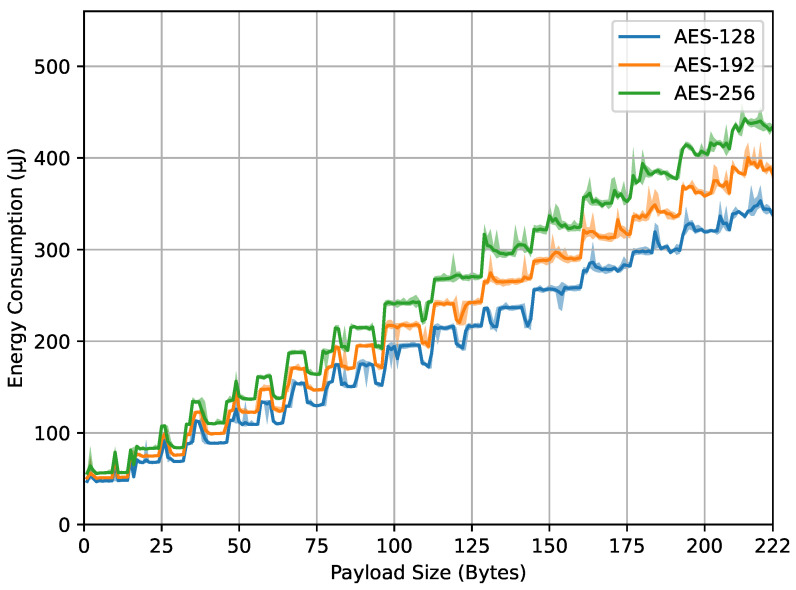
encrypt_payload() energy.

Figure 7 shows the energy consumption measured on the ED when executing the encrypt_payload() function with varying payload sizes and AES key sizes. The x-axis indicates the payload sizes in bytes, while the y-axis represents the energy consumption in microjoules. The different curves in the graph correspond to the different AES key sizes used. It can be observed first that energy consumption increases with larger AES key sizes and payload sizes, which was expected behavior. Larger AES key sizes (192 and 256 bits) consume energy more than the smaller size (128 bits) across all tested payload sizes. As explained in Section 3.4, the reason is the extra encryption rounds required for larger keys, which translate to more computation time. Similarly, the trend of increased energy consumption with larger payload sizes is due to the increased processing required to encrypt larger amounts of data. Interestingly, the relationship between payload size and energy consumption is not linear. There are specific payload sizes where energy consumption surges for all three AES key sizes. The reasons for these significant increases require further investigation but are likely due to the activity of other parts of the electronic circuitry on the ED board.

**Figure 8:**
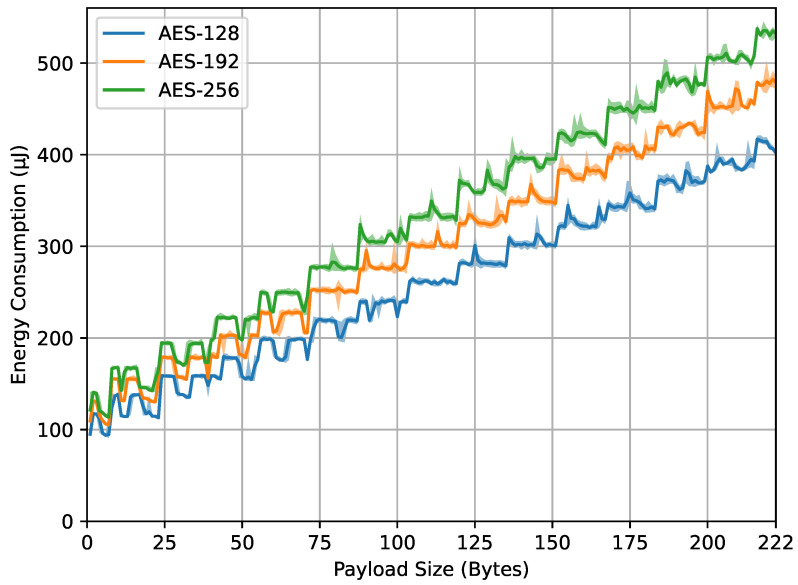
compute_mic() energy.

Figure 8 also represents energy consumption, but for the compute_mic() function. The axes and different curves have the same meaning as in Figure 7. The behavior in compute_mic() is such that the total data passed to this function consist of the payload data compounded with an overhead size of 9 bytes. For example, a payload size of 7 bytes (as represented on the x-axis), plus a 9-byte overhead, results in a total data size of 16 bytes. The observations indicate that, as in Figure 7, Figure 8 shows that larger AES key sizes continue to consume more energy. This reflects the increased computational complexity associated with these larger keys, as is the case with larger payload sizes. In addition, mirroring the behavior of encrypt_payload() in Figure 7, some points of the payload appear as spikes, possibly for the same reasons.

### 6.2. Measured Computation Time

**Figure 9:**
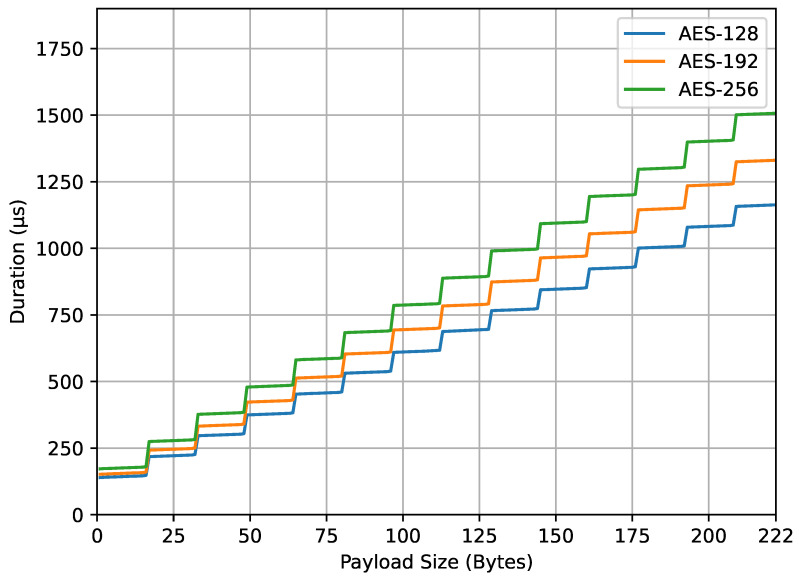
encrypt_payload() duration.

Figure 9 illustrates the computation time of the encrypt_payload() function. Unlike previous graphs, the y-axis in this figure represents duration in microseconds. It was found that the use of larger AES key sizes (192 and 256 bits) requires longer computation times compared to the smaller AES key size (128 bits). This can be attributed to the higher number of rounds needed for encryption with larger key sizes. It was also found that the computation time increases as the payload size expands due to the increased computational load involved in encrypting larger payloads. Additionally, a significant increase in computation time is observed when the payload size exceeds a multiple of the AES block size (16 bytes). For example, at payload sizes of 17, 33, 49 bytes, and so on, the surplus byte necessitates an additional block by padding. The number of encryption computation rounds corresponds to the number of data blocks. For instance, payload sizes from 17 to 32 bytes fall into the same block number category (2 blocks). This data padding technique, used to fulfill the data block requirement, provides the graph a stair-step appearance.

**Figure 10:**
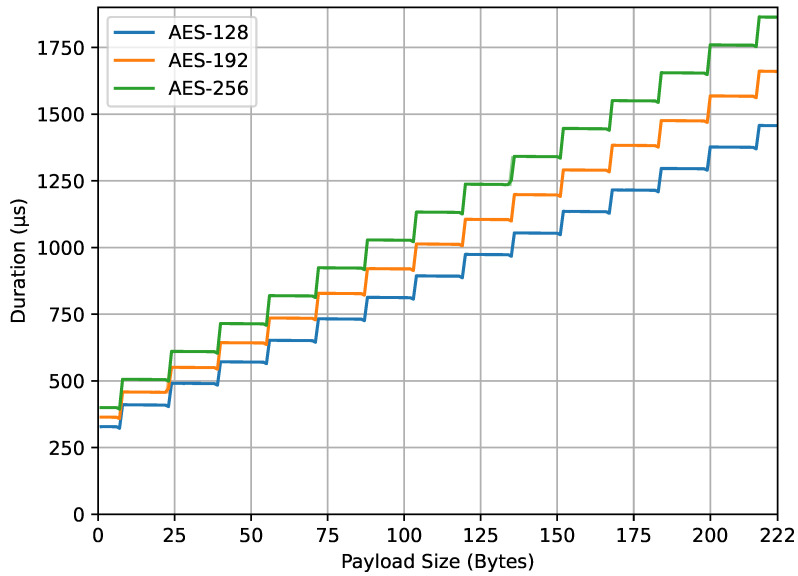
compute_mic() duration.

Figure 10 displays the computation time of the compute_mic() function, with the same axes as shown in Figure 9. The graph demonstrates a trend of increasing computation time as both the AES key size and payload size increase. The shape of the graph resembles a stairway, similar to encrypt_payload() in Figure 9, due to the padding of data added to the payload to fulfill the block requirements. Similar to Figure 8, the higher values compared to the encrypt_payload() function can be attributed to an overhead of 9 bytes added to the payload data before they are passed to the compute_mic() function. Part of this increase can also be ascribed to the preparation and encryption of the initial block $B_{0}$, as discussed in Section 3.6. An interesting observation from this figure is the occurrence of very small dips at payload sizes of 7, 23, 39 bytes, and so forth (which equate to real sizes of 16, 32, 48 bytes, and so forth) before they rise again. This is because the AES-CMAC task within the compute_mic() function does not require additional padding as the real size of the data is a multiple of 16 bytes or 128 bits.

From the four result graphs, we examined the values of energy consumption and computation time at the maximum payload size of 222 bytes for different AES key sizes (128, 192, and 256 bits). The energy consumption values for encrypt_payload(), as shown in Figure 7, are 337, 382, and 443 microjoules, while, for compute_mic(), in Figure 8, they are 402, 478, and 532 microjoules. The computation times for encrypt_payload(), in Figure 9, are 1163, 1330, and 1507 microseconds, and, for compute_mic(), in Figure 10, they are 1457, 1660, and 1863 microseconds. The ratios of these values, when using longer AES key sizes (192 and 256 bits) compared to the LoRaWAN standard (128 bits), are shown in Table 1. We observed that the increased cost ratio from both measurements, energy consumption and duration, are 1.13, 1.14, 1.19, and 1.14 for AES-192 compared to AES-128, and 1.31, 1.30, 1.32, and 1.28 for AES-256 compared to AES-128.

### 6.3. Regression Analysis

To further our understanding of the impact of different AES key sizes on the encryption duration and MIC computation, regression analysis was conducted on the collected data. The regression models for each key size provide insights into the computation time variations influenced by the key lengths.

**Figure 11:**
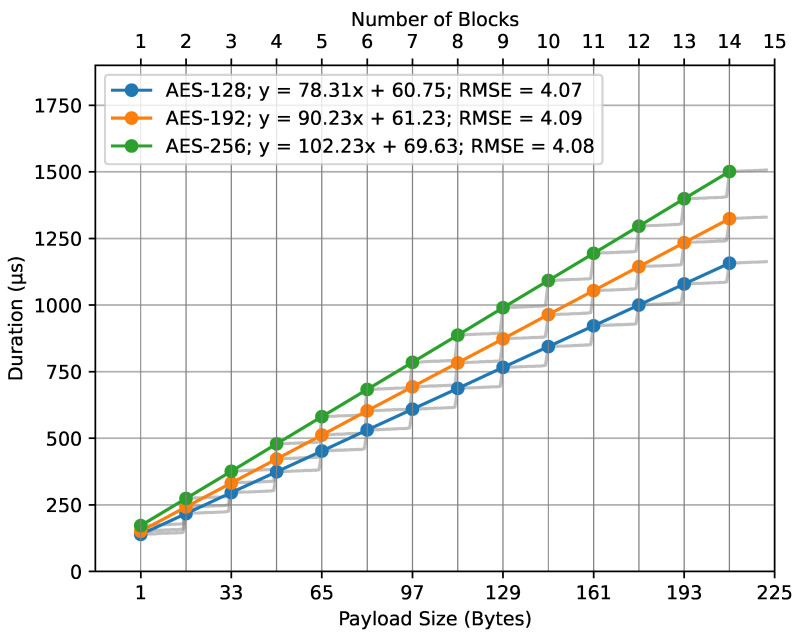
encrypt_payload() duration regression.

Figure 11 presents the data plot with fit lines for the AES key sizes. The fit line equations, where *x* represents the number of blocks, are as follows: for AES-128, y=78.31x+60.75; for AES-192, y=90.23x+61.23; and, for AES-256, y=102.23x+69.63. All these models have an approximate RMSE of 4. The number of blocks is computed using Equation (6). The lower x-axis represents the payload size in bytes, which is mapped to the number of blocks on the upper x-axis.

**Figure 12:**
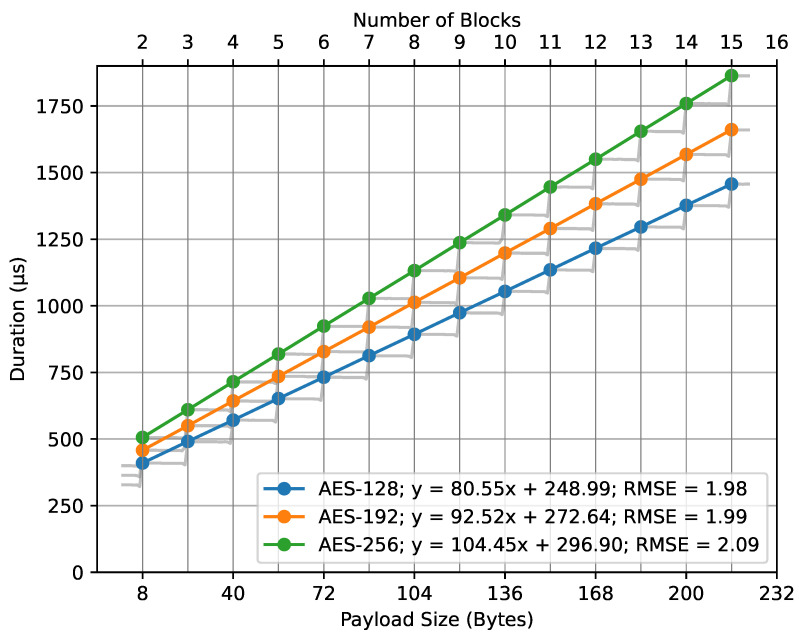
compute_mic() duration regression.

Similarly, Figure 12 showcases the fit lines for the AES key sizes in relation to the compute_mic() function. Here, where *x* represents the number of blocks, the fit line equations are as follows: for AES-128, y=80.55x+248.99; for AES-192, y=92.52x+272.64; and, for AES-256, y=104.45x+296.90. All these models have an RMSE value of approximately 2. The number of blocks is determined using Equation (10). The lower x-axis represents the payload size in bytes, which corresponds to the number of blocks displayed on the upper x-axis.

In both cases, the number of blocks was mapped to the payload size in bytes, as described in our mathematical models (Equations (6) and (10)). The observed regression lines from the figures support the basic principles of the mathematical model, specifically Equation (7). The close alignment between empirical data and the theoretical model is emphasized by the low RMSE values. These values highlight the accuracy of our regression models, suggesting minimal deviations between observed and predicted outcomes. Essentially, the low RMSEs enhance our trust in the models.

**Figure 13:**
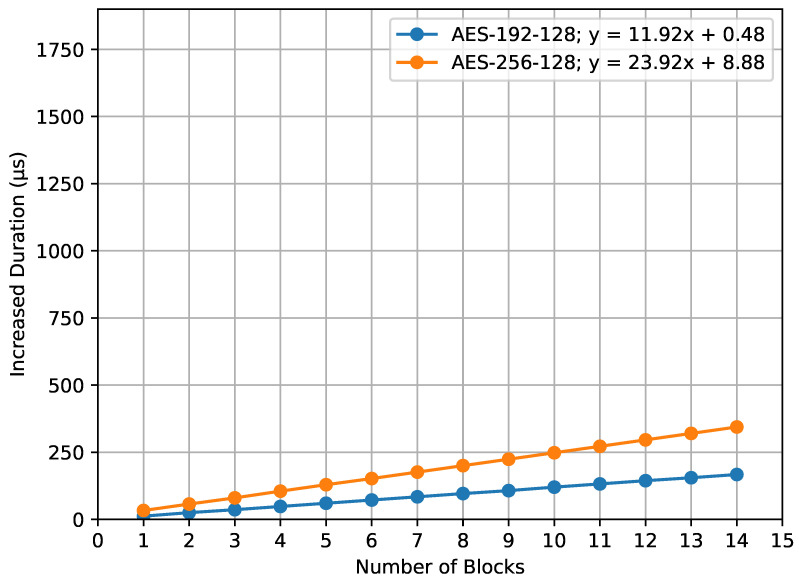
encrypt_payload() increased duration.

Figure 13 depicts the increased duration for the encrypt_payload() function with respect to varying AES key sizes. The graph portrays two distinct lines, representing the increase in computation time for AES-192 compared to AES-128 (denoted as AES-192-128) and for AES-256 compared to AES-128 (denoted as AES-256-128). The x-axis of the graph represents the number of blocks. Specifically, the equation for the AES-192-128 line is provided by y=11.92x+0.48, while the AES-256-128 line is represented by y=23.92x+8.88.

**Figure 14:**
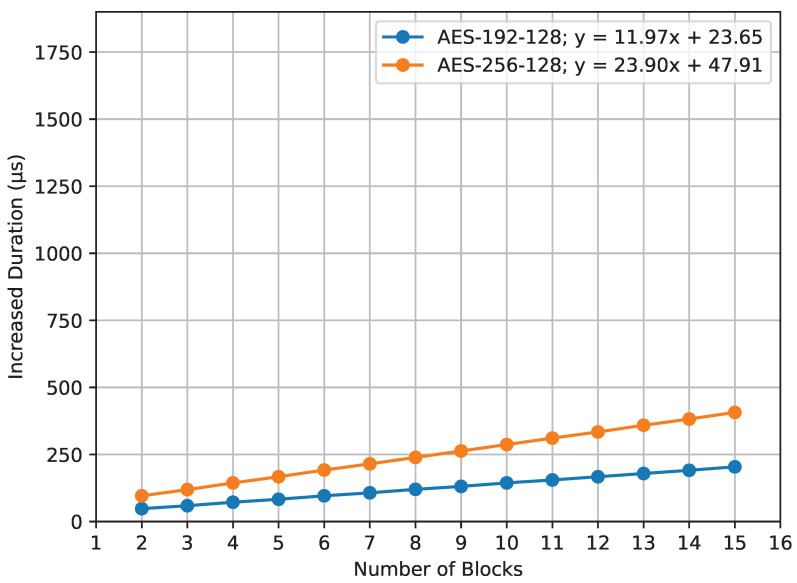
compute_mic() increased duration.

Figure 14 visualizes the increased computation time for the compute_mic() function under the influence of different AES key sizes. For AES-192-128, the duration increase is described by the equation y=11.97x+23.65, and, for AES-256-128, it is y=23.90x+47.91.

A clear observation from both graphs is the mild inclines of the plotted lines showing increased durations. This gentle rise shows that, while using larger AES key sizes does add some time, the added time for each block is small and manageable. The slight increases in the lines highlight that AES encryption remains efficient, meaning that using larger key sizes does not make the overall processing times grow too much.

### 6.4. Comparison to the Cost of Radio Communications

Furthermore, for a payload size of 222 bytes, when comparing AES-256 to AES-128, the increased computation time is 750 microseconds and the increased energy consumption is 236 microjoules. Notably, there is a direct correlation between the results of energy consumption and computation time. Even though these increases in energy and time seem significant, it is interesting to put them in perspective and compare them to a major cause for energy consumption on the ED, namely the actual radio communications. As we calculated in Section 3.7, the time on air of a frame carrying a payload of 222 bytes, using SF7, a bandwidth of 125 kHz, and a coding rate of 4/5, amounts to around 368.9 ms, and we measured the energy spent by the ED during that period to around 183.7 μJ. These observations are summarized in Table 2 to highlight that, in terms of time on air costs, the increases represent just 0.2% and 0.13%, respectively. This emphasizes that the extra costs of using longer AES key sizes are small when compared with the main energy and time demands during time on air. Increasing the AES key size is, therefore, unlikely to significantly reduce the lifetime of end devices while enhancing the security of communications.

Summing up, our results indicate that, while both energy consumption and computation time increase with the use of larger AES key sizes and increased payload sizes, these increases are negligible when compared to time on air costs.

Data from Figure 6, Figure 7 and Figure 8 highlight the feasibility and efficiency of using longer AES keys in LoRaWAN communication. While there is a noticeable rise in energy and time with larger key sizes, this increase is trivial in terms of the overall cost.

A notable pattern was observed at payload sizes corresponding to multiples of 16 bytes, as detailed in Figure 10. At these points, no extra padding was needed, showcasing that the encryption process behavior aligns well with its theoretical foundations.

To conclude, our analysis demonstrates that, while employing larger AES key sizes in LoRaWAN brings about minor overheads, the overall efficiency remains largely unaffected. This sets the stage for a deeper exploration into the implications and potential applications of our findings.

## 7. Discussion

Our research aimed to explore the energy consumption and computational time duration associated with employing larger AES key sizes in LoRaWAN communication, and how this may impact the overall efficiency of the system. Our findings indicate that, while larger key sizes do result in slightly increased energy and computational costs, these increments are minimal when compared to the most resource-consuming tasks, specifically those during time on air.

However, while our study provides valuable insights into the implications of using larger AES keys in LoRaWAN communication, it is essential to note certain limitations. Specifically, our measurements of energy consumption during the processing phase were not isolated entirely from interference caused by other components of ED. This may introduce a degree of imprecision in our energy consumption results. However, we have greater confidence in our computational time duration measurements as they offer a more accurate representation unaffected by these external influences.

Given this, an important aspect of our research is that it aligns with the prevalent trend towards improving security in IoT and wireless communication networks. The fact that the extra costs of using longer AES keys in the encryption functions do not significantly affect the overall operation of the system provides evidence to support the transition towards longer AES keys for improved security in LoRaWAN and other IoT communication systems that use AES encryption, such as IEEE 802.15.4 [33], ZigBee [39], and Sigfox [40].

In light of our findings, to facilitate the adoption of longer AES keys in LoRaWAN, it is proposed to update the firmware of ED to support the longer AES encryption function and to integrate the new longer AES key. Concurrently, the software of both NS and AS should be modified to accommodate the longer AES encryption function and to update the database with the new longer AES key of ED. This coordinated approach promises a seamless transition and enhanced network security.

On a broader note, in the current LoRaWAN system landscape, updating the root key requires a firmware modification for ED, which is a labor-intensive process and makes keeping the key up to date challenging. Recognizing this limitation, our future research efforts will explore the potential benefits of utilizing public key cryptography as a mechanism to facilitate more seamless and frequent updates to these longer root keys. By innovating ways to simplify and expedite root key renewals, we hope to significantly enhance the security resilience of the LoRaWAN communication system.

Looking ahead, as we move further into the digital age, the evolution of computing power is certain. Quantum computing and the continual growth in classical computing capabilities pose an approaching threat to our current cryptographic standards. Adopting larger AES keys now proactively addresses this future challenge. By enhancing encryption robustness, we not only ensure the security of present-day IoT communications but also prepare our systems for potential decryption threats in the coming decades. This forward-thinking approach ensures that IoT devices and networks remain resilient against malicious activities, even as potential opponents gain access to more powerful computational tools.

In conclusion, the security of IoT communication is a critical concern, especially in the context of LoRaWAN due to its wide range of applications. The results of this study demonstrate that, while employing larger AES keys for encryption does result in some increased costs, these increments are minimal compared to the total cost. This suggests that the implementation of larger AES keys is a viable and potentially beneficial approach for enhancing security in LoRaWAN communication.

## Figures and Tables

**Table 1:** Comparison of increased ratios to AES-128 of payload 222 bytes.

Function	Energy		Duration	
	AES-192	AES-256	AES-192	AES-256
encrypt_payload()	1.13	1.31	1.14	1.30
compute_mic()	1.19	1.32	1.14	1.28

**Table 2:** Comparison of the additional cost of encryption and radio transmission for the maximum payload size (222 bytes).

	Energy	Computation Time
Additional encryption cost	236 μJ	750 μs
Radio transmission	183.7 m J	368.9 m s
Ratio	0.13%	0.2%

## Data Availability

Data are contained within the article.

## Abbreviations

The following abbreviations are used in this manuscript:

ABP	Activation By Personalization
ADR	Adaptive Data Rate
AES	Advanced Encryption Standard
AS	Application Server
CA	Certificate Authority
CCM*	Counter with CBC-MAC*
CMAC	Cipher-based Message Authentication Code
ED	End Device
GW	Gateway
IoT	Internet of Things
JS	Join Server
LPWAN	Low Power Wide Area Network
MIC	Message Integrity Code
NS	Network Server
OTAA	Over-The-Air Activation
PKI	Public Key Infrastructure
SF	Spreading Factor
ToA	Time on Air
